# Editorial: Omnichannel Customer Behavior: New Questions in the Age of Agility

**DOI:** 10.3389/fpsyg.2021.746147

**Published:** 2021-09-13

**Authors:** Eva Reinares-Lara, Cristina Olarte-Pascual, Jorge Pelegrín-Borondo, Gwenaëlle Oruezabala

**Affiliations:** ^1^Department of Business Administration, Universidad Rey Juan Carlos, Madrid, Spain; ^2^Department of Marketing, University of La Rioja, Logroño, Spain; ^3^Department of International Business, University of Nantes, Nantes, France

**Keywords:** omnichannel, customer behavior, retail, webrooming, adoption of technologies

This Research Topic aims to shed light on omnichannel management, one of the most important retail revolutions in recent years. “Omnishopper” customers place higher demands on information and communication technology in terms of the retail formats through which consumers can engage with a business. Furthermore, in addition to traditional physical and online shops, new channels, and touchpoints such as mobile phones, social media, and wearables (e.g., smartwatches) have transformed the consumer shopping journey process (e.g., Juaneda-Ayensa et al., 2016).

Previous research on the omnichannel retailing topic has shown that brands are increasingly agile. Companies and retailers strive to ensure consistent communication across the various channels and touchpoints customers may wish to use in order to provide them with a superior shopping experience. Nevertheless, the related topic of the relationship between omnichannel customer behavior and companies' agile strategies gives rise to new research opportunities and concerns for the marketing discipline and the social sciences in general. There is a need for new theoretical frameworks and insights into empirical applications that could trigger interactions among researchers and practitioners from a variety of fields, such as marketing, e-commerce, strategy, or information systems, in which consumer behavior and psychology play a central role.

The present collection of six articles builds upon previous research and brings together literature on shopper behavior. Although the papers examine the impact of different variables—endogenous factors or externalities—on consumer behavior, they have a common thread: the omnichannel retailing context. They are also linked by the functional focus of integrating technologies into the related business field, thereby demonstrating the required agility in the strategies that companies deploy.

Gutiérrez and García highlight omnichannel strategy and consumer behavior in distribution channels with a specific focus on trends in the ophthalmology sector. The authors use the Delphi method to identify trends and problems and show that the arrival of omnichannel distribution is inevitable in this sector. Their findings confirm that companies are undergoing a major transformation from a rigid traditional distribution model to a more complex and flexible model due to the entry of new online intermediaries with service integration models that also shorten traditional distribution channels.

Kim et al. discuss the heterogeneous influence of gamification and novelty-seeking traits on consumers' repurchase intention in the omnichannel retailing context. Their study confirms that omnichannel retailers have to become more agile by exploring new services such as gamification, which is known to enhance customer loyalty, positive word-of-mouth, and engagement. However, they argue that platform gamification will not yield results with all consumers. Consequently, they suggest that practitioners approach the implementation of game mechanics in non-game contexts using an opt-in rather than compulsory option.

Paz and Delgado review and analyze models related to the influence of sales atmospherics on consumer behavior in shopping contexts that combine physical sales settings with new digital sales atmospheres. They provide a table summarizing the main published studies testing the validity of the S-O-R model in the context of online outlets, in which stores cease to be physical spaces and the shopping experience is augmented by virtual settings.

Kleinlercher et al. examine the antecedents of webrooming in omnichannel retailing. Their conceptual framework, based on Anticipated Utility Theory, allows them to demonstrate that the utility customers expect to gain from using a physical store vs. an online store to make a purchase can be predicted by four groups of antecedents: psychographic variables, shopping motivations, channel-related variables, and product-related variables. They also find a positive association between webrooming and customer spending.

Lawry and Bhappu measure consumer engagement in omnichannel retailing through the application of a mobile in-store experience (MIX). Drawing insights from Activity Theory, they develop a nine-item index to quantitatively measure the shopping experience, including purchase activities and retail services. They suggest that retailer agility could consist of giving consumers the autonomy to co-create personalized shopping experiences as active participants within an omnichannel retail services cape while purchasing merchandise in brick-and-mortar stores.

Liang et al. detail how smog affects customer channel choice—online or offline—for purchasing fresh food. Building on the idea that an adverse external environment might cause gradual change in people's habits and shopping behaviors, they confirm that smog levels have a significant positive effect on online channel purchasing. Retailer agility might thus consist of adjusting channel strategies to account for harsh external conditions, taking into consideration that variations in product pricing and consumer trends toward healthy eating would strengthen the positive association between smog and online purchasing.

## Author Contributions

All authors listed have made a substantial, direct and intellectual contribution to the work, and approved it for publication.

## Conflict of Interest

The authors declare that the research was conducted in the absence of any commercial or financial relationships that could be construed as a potential conflict of interest.

## Publisher's Note

All claims expressed in this article are solely those of the authors and do not necessarily represent those of their affiliated organizations, or those of the publisher, the editors and the reviewers. Any product that may be evaluated in this article, or claim that may be made by its manufacturer, is not guaranteed or endorsed by the publisher.
